# Book Review: The Handbook of Neurosurgery 8th Edition by Mark S. Greenberg: A Review of its Use in Clinical Practice

**DOI:** 10.3389/fsurg.2017.00053

**Published:** 2017-09-20

**Authors:** Syed I. Khalid, Owoicho Adogwa

**Affiliations:** ^1^Department of Neurosurgery, Rush University Medical Center, Chicago, IL, United States

**Keywords:** neurosurgery, clinical resources, book review, neurology, neurosciences

The Handbook of Neurosurgery 8th Edition by Mark S. Greenberg is an excellent reference for rapid review when a decision about a clinical situation requires a quick review of relevant and adequately detailed information. In this way, The Handbook of Neurosurgery is much more useful in most clinical situations than many of the multi-volume neurosurgery texts available today. The updated version feels significantly cleaner and meaningfully better organized than previous versions, with a new color coding organization that helps quickly navigate otherwise very complex information. This text seems to have pretty much everything you need, with very little, if anything, that you don’t need—covering, with appropriate, depth and breadth, the field of neurosurgery from the perspective of anatomy, physiology, differential diagnosis, and the current principles of non-surgical and surgical management. The text covers both pediatric through geriatric age ranges, covering nearly all inherited, developmental, and acquired neurological disorders. The Handbook of Neurosurgery 8th Edition by Mark S. Greenberg also covers topics and illnesses more commonly treated by neurointensivists and neurologists, including adequately detailed coverage of topics surrounding dementia, motor neuron diseases, parkinsonism, and multiple sclerosis. All in all, Greenberg’s Handbook of Neurosurgery 8th Edition is comprehensive and conveniently compact and is a must-have reference for anyone working in the neurosurgery, neurology, neurointensive care, and the neurosciences.

## Author Contributions

SK and OA: comprehensive use of book, notes during use, drafting review, and editing submission.

## Conflict of Interest Statement

The authors declare that the research was conducted in the absence of any commercial or financial relationships that could be construed as a potential conflict of interest.

